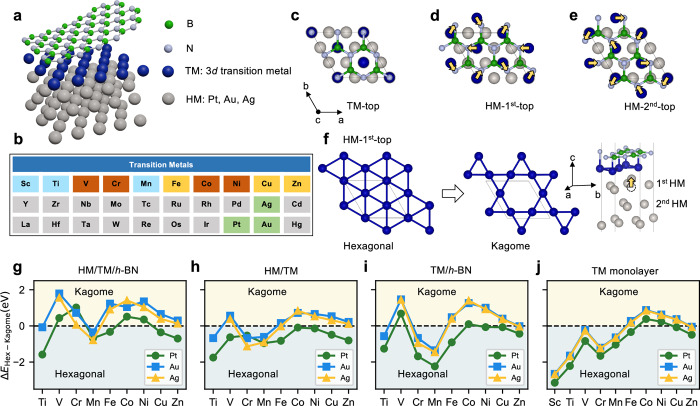# Publisher Correction: Kagomerization of transition metal monolayers induced by two-dimensional hexagonal boron nitride

**DOI:** 10.1038/s41467-024-51436-0

**Published:** 2024-08-28

**Authors:** Hangyu Zhou, Manuel dos Santos Dias, Youguang Zhang, Weisheng Zhao, Samir Lounis

**Affiliations:** 1 Peter Grünberg Institut and Institute for Advanced Simulations, Forschungszentrum Jülich & JARA, 52425 Jülich, Germany; 2 School of Electronic and Information Engineering, Beihang University, Beijing, 100191 China; 3 Fert Beijing Institute, School of Integrated Circuit Science and Engineering, Beihang University, Beijing, 100191 China; 4 Shenyuan Honors College, Beihang University, Beijing, 100191 China; 5 Faculty of Physics, University of Duisburg-Essen and CENIDE, 47053 Duisburg, Germany; 6 Scientific Computing Department, STFC Daresbury Laboratory, Warrington, WA4 4AD United Kingdom

**Keywords:** Spintronics, Information storage, Two-dimensional materials

Correction to: *Nature Communications* 10.1038/s41467-024-48973-z, published online 6 June 2024

In this article the wrong version of Fig. 1 was used, in which y-axis labels were duplicated across panels 1h–j; the figure should have appeared as shown below. In addition, in the sentence beginning ‘Figure 3g shows the values of…’, the term ‘Figure 3g’ should have read ‘Figure 3h’. The original article has been corrected.