# Supplementary material for: Kagomerization of transition metal monolayers induced by two-dimensional hexagonal boron nitride
Source: Nat Commun. 2024 Jun 6;15:4854. doi: 10.1038/s41467-024-48973-z (PMC11156855; doi:10.1038/s41467-024-48973-z)
Supplement: Supplementary file 1 — Supplementary Information [file 41467_2024_48973_MOESM1_ESM.pdf]

Supplementary Information: Kagomerization of transition metal monolayers induced by two-dimensional hexagonal boron nitride

Hangyu Zhou[1,2,3,4,*], Manuel dos Santos Dias[1,5,6], Youguang Zhang[2], Weisheng Zhao[3,*], and Samir Lounis[1,5,*]

[1]Peter Grünberg Institut and Institute for Advanced Simulations, Forschungszentrum Jülich & JARA, 52425 Jülich, Germany

[2]School of Electronic and Information Engineering, Beihang University, Beijing 100191, China

[3]Fert Beijing Institute, School of Integrated Circuit Science and Engineering, Beihang University, Beijing 100191, China.

[4]Shenyuan Honors College, Beihang University, Beijing 100191, China

[5]Faculty of Physics, University of Duisburg-Essen and CENIDE, 47053 Duisburg, Germany

[6]Scientific Computing Department, STFC Daresbury Laboratory, Warrington WA4 4AD, United Kingdom

[*]h.zhou@fz-juelich.de; weisheng.zhao@buaa.edu.cn; s.lounis@fz-juelich.de

Supplementary Note 1: Kagomerization

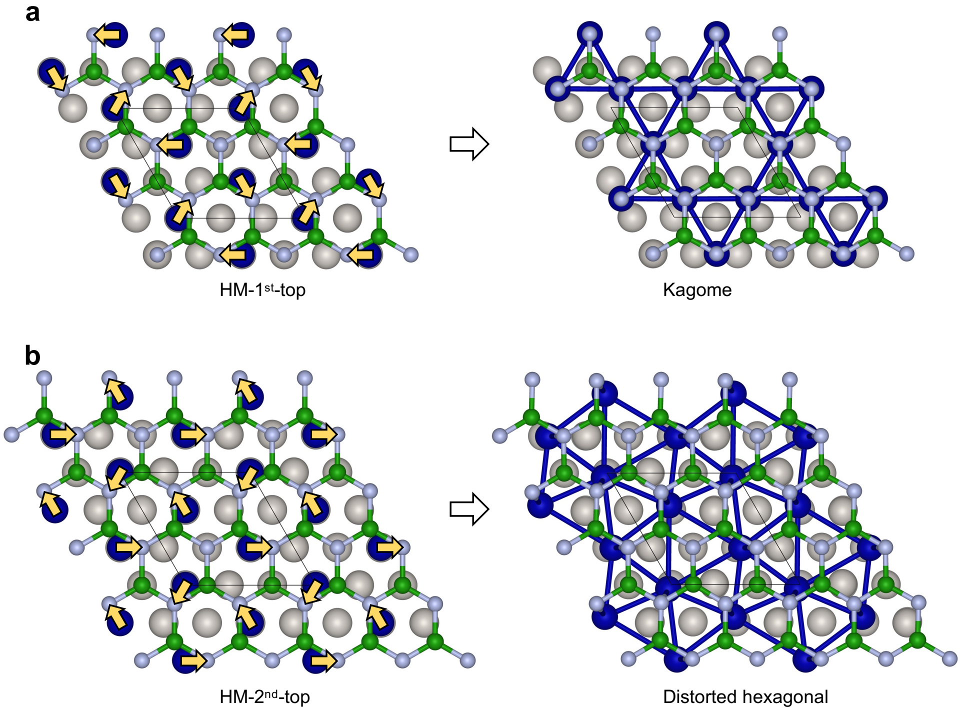

Figure S1: Motion of the Co atoms on Pt substrate driven by *h*-BN. a Kagome lattice relaxed from HM-1st-top stacking. **b** Distorted hexagonal lattice relaxed from HM-2nd-top stacking.

Fig. S1 shows an example of how the kagome and distorted hexagonal lattices form in the presence of *h*-BN. The initial structures are shown on the left (Fig. S1a for HM-1st-top and Fig. S1b for HM-2nd-top), and the optimized structures obtained from them are shown on the right (Fig. S1a for kagome and Fig. S1b for distorted hexagonal).

In Fig. S2, we summarize various combinations of 3*d* transition metals and substrates, indicating whether kagomerization can occur spontaneously and be the ground state. This table provides further details for Fig. 1b in the main text. In some systems, kagomerization can not

occur spontaneously starting from the HM-1st-top configuration. Therefore we implemented a kagome lattice structure for the TM layer and performed geometry optimization. We present the distances between *h*-BN and the TM layer, the buckling of *h*-BN layer, and the distances between the TM layer and the elevated HM atom in the kagomerized structures in Fig. S3a, b and c, respectively. Upon examining the distances between *h*-BN and the TM layer (Fig. S3a), we observe that in the kagomerized structures, *h*-BN exhibits physisorption in cases involving Cu and Zn, while chemisorption occurs in the remaining cases. The buckling is insignificant when *h*-BN is physisorbed on the surfaces, as shown in Fig. S3b.

In Table S1, we consider the free-standing monolayers and release the epitaxial constraint of matching the lattice constant of the substrate. We find that the total energy differences always favor the hexagonal over the kagome structure at the respective theoretically optimized lattice constants.

	Ground state			Spontaneity		
	Pt	Au	Ag	Pt	Au	Ag
Sc	✗	✗	✗	✗	✗	✗
Ti	✗	✗	✗	✗	✗	✗
V	✓	✓	✓	✗	✓	✓
Cr	✓	✓	✓	✗	✓	✓
Mn	✗	✗	✗	✗	✓	✓
Fe	✗	✓	✓	✗	✓	✓
Co	✓	✓	✓	✓	✓	✓
Ni	✓	✓	✓	✓	✓	✓
Cu	✗	✓	✓	✗	✓	✓
Zn	✗	✓	✓	✗	✓	✓

Figure S2: Summary of results for the kagomerization. We show whether the kagome structure is the ground state structure and whether it forms spontaneously (in the sense defined in the text) for the considered combinations of 3d transition metals and substrates.

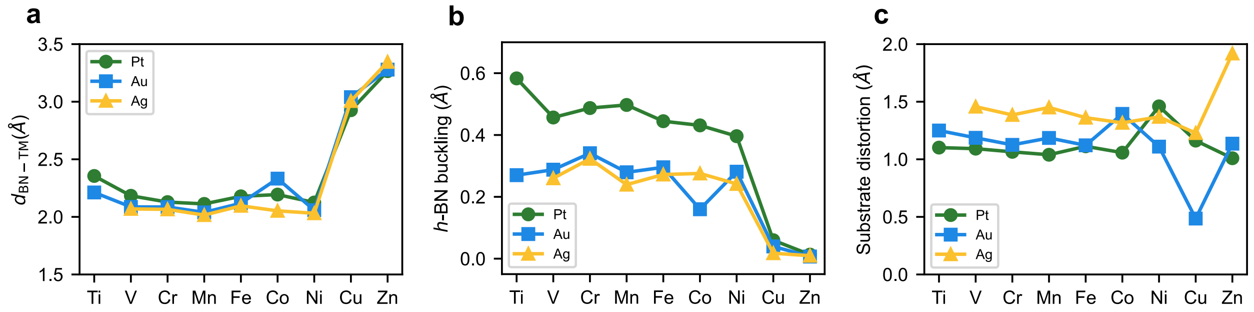

Figure S3: Structural parameters for kagomerized structures. a Distances between h-BN and the TM layer. **b** Buckling of h-BN layer. **c** Distances between the TM layer and the elevated HM atom.

	Sc	Ti	V	Cr	Mn	Fe	Co	Ni	Cu	Zn
$\Delta E_{\mathrm{Hex-Kagome}}$	-1.51	-2.33	-2.38	-1.72	-1.42	-2.09	-1.97	-1.93	-1.41	-1.04
a_{Hex}	5.43	4.63	4.23	4.58	4.50	4.16	4.05	4.08	4.19	4.41
a_{Kagome}	6.00	5.04	4.56	5.14	4.96	4.74	4.51	4.57	4.71	5.00

Table S1: Energy differences $\Delta E_{\mathrm{Hex-Kagome}}$ (eV) between the freestanding hexagonal and kagome structures of the TM monolayer with relaxed lattice constants (Å).

Supplementary Note 2: Band structure

Here we present the band structures of additional structures beyond those shown in the main text.

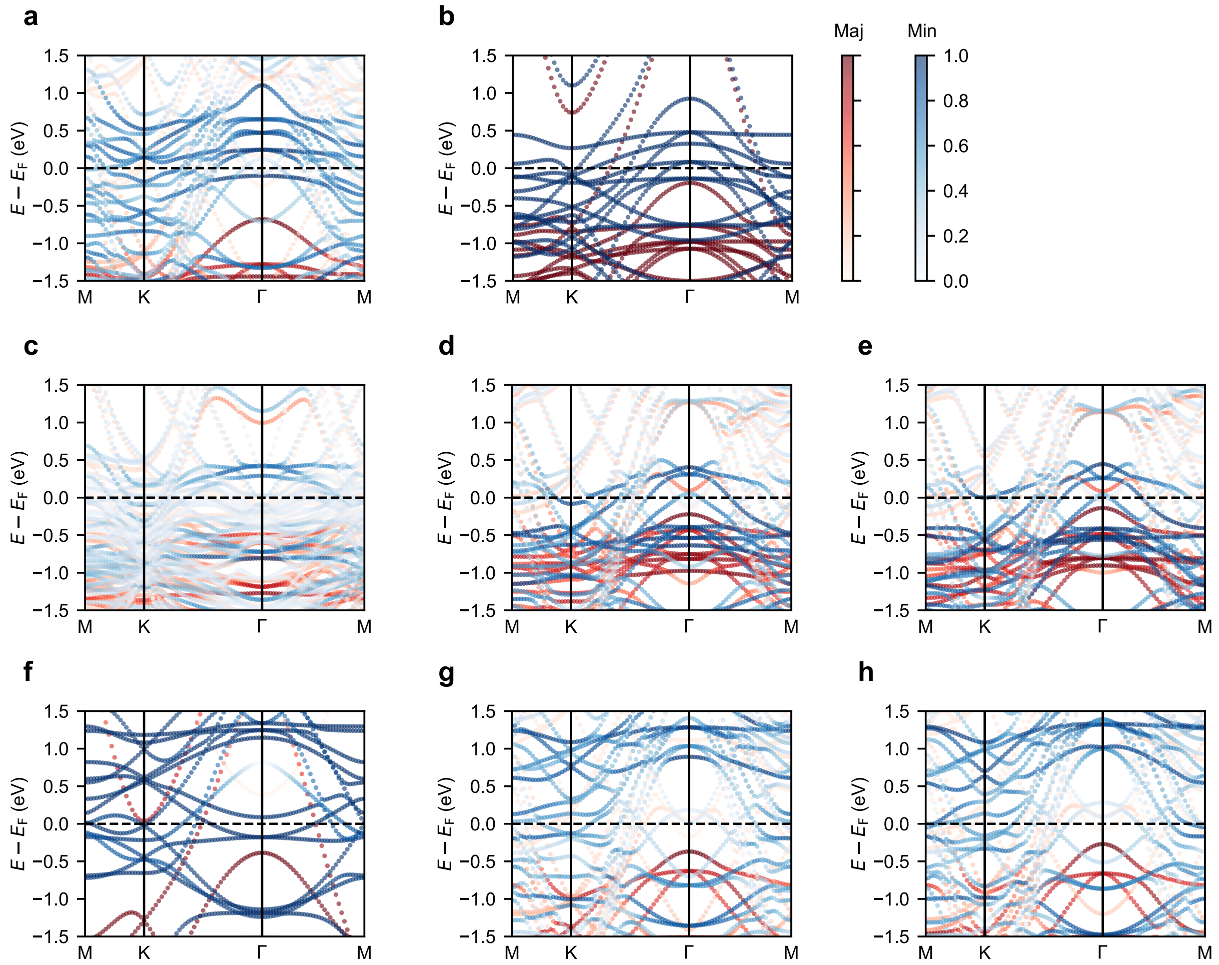

Figure S4: Spin-resolved projected band structures of kagomerized structures. Spin- and FM-projected band structures for: **a** Au/Co/*h*-BN; **b** isolated Ni monolayer kagome lattice; **c** Pt/Ni/*h*-BN; **d** Au/Ni/*h*-BN; **e** Ag/Ni/*h*-BN; **f** isolated Fe monolayer kagome lattice; **g** Au/Fe/*h*-BN; **h** Ag/Fe/*h*-BN.

TM	Fe		Co			Ni		
HM	Au	Ag	Pt	Au	Ag	Pt	Au	Ag
without *h*-BN	3.09	3.08	2.01	1.96	1.93	0.81	0.67	0.63
with *h*-BN	2.78	2.73	1.84	1.88	1.78	0.58	0.54	0.49

Table S2: Spin moment (in μ_{B}) for the transition metal (TM) monolayers on various heavy-metal (HM) surfaces, without *h*-BN (hexagonal structure) and with *h*-BN (kagome structure).

Supplementary Note 3: Spin moments

Here we show the spin moments for the HM/TM (hexagonal, labelled w/o-BN-Hex) and for the HM/TM/*h*-BN (kagome, labelled w/-BN-Kagome) structures in Table S2.

Supplementary Note 4: Decomposition of the kagomerized structures

To explore the impact of kagomerization and *h*-BN respectively, we decompose the kagomerized structures. Therefore, we have four structural configurations. The first configuration is the hexagonal HM/TM structure without *h*-BN (w/o-BN-Hex), as shown in Fig. S5a. Then we manually constructed the HM/TM with *h*-BN overlay while maintaining the TM layer in hexagonal arrangement (w/-BN-Hex), as shown in Fig. S5b. In this case, geometry optimization has been done for the structures with Au or Ag substrates but fixing the lateral coordinates of the TM atoms and *h*-BN. However, for the structures with Pt substrate, fixing lateral coordinates leads to a distance between *h*-BN and the TM layer exceeding 3Å after relaxation. Therefore, we manually constructed the structures with Pt substrates, taking the relaxed z-direction distance of the kagome Pt/TM/*h*-BN as a reference. Additionally, from the kagome structure with *h*-BN (w/-BN-Kagome), we directly remove the *h*-BN overlayer to obtain another configuration (w/o-BN-Kagome), as shown in Fig. S5c.

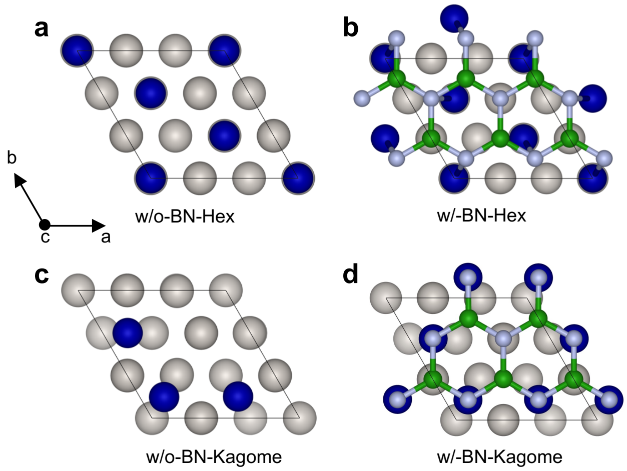

Figure S5: Four structural configurations. a Hexagonal HM/TM structure without *h*-BN (w/o-BN-Hex). **b** HM/TM with *h*-BN overlay and hexagonal TM layer (w/-BN-Hex). **c** HM/TM without *h*-BN overlay, but with kagome TM layer (w/o-BN-Kagome). **d** Relaxed HM/TM/*h*-BN with kagome TM layer (w/-BN-Kagome).

Supplementary Note 5: Dependence of the micromagnetic parameters on the spatial cutoff

To have a better understanding of how the magnetic interactions change, we present here the stiffness-tensor and spiralization-tensor element as a function of the cut-off radius, as shown in Fig. S6 and Fig. S7. In Fig. 3g and h of the main text, the cut-off radius for the real space summations is $8a$ (Pt/Co, Au/Co, Ag/Co, Pt/Ni, Au/Ni and Ag/Ni systems) or $14a$ (Au/Fe and Ag/Fe systems). As we see, the stiffness-tensor elements of Au/Fe and Ag/Fe systems undergo severe oscillations, prompting the use of a larger cut-off radius instead of $8a$.

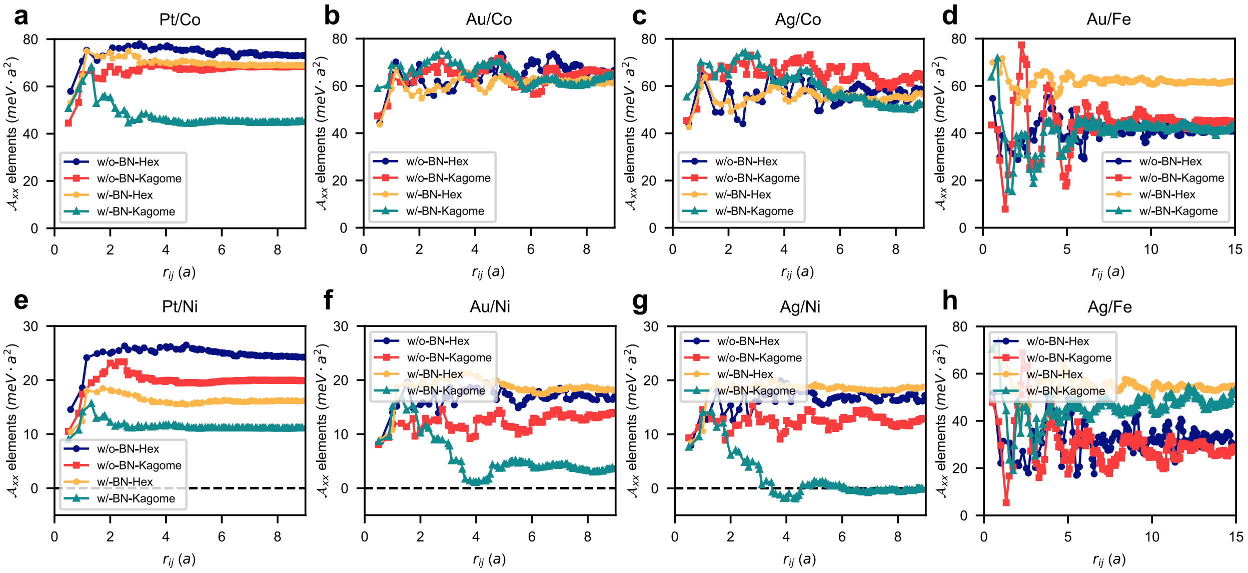

Figure S6: Exchange-stiffness-tensor element $\mathcal{A}_{xx}$ as a function of the cut-off radius. Panel **a-h** are the results for four structural configurations of Pt/Co, Au/Co, Ag/Co, Au/Fe, Pt/Ni, Au/Ni, Ag/Ni, and Ag/Fe systems.

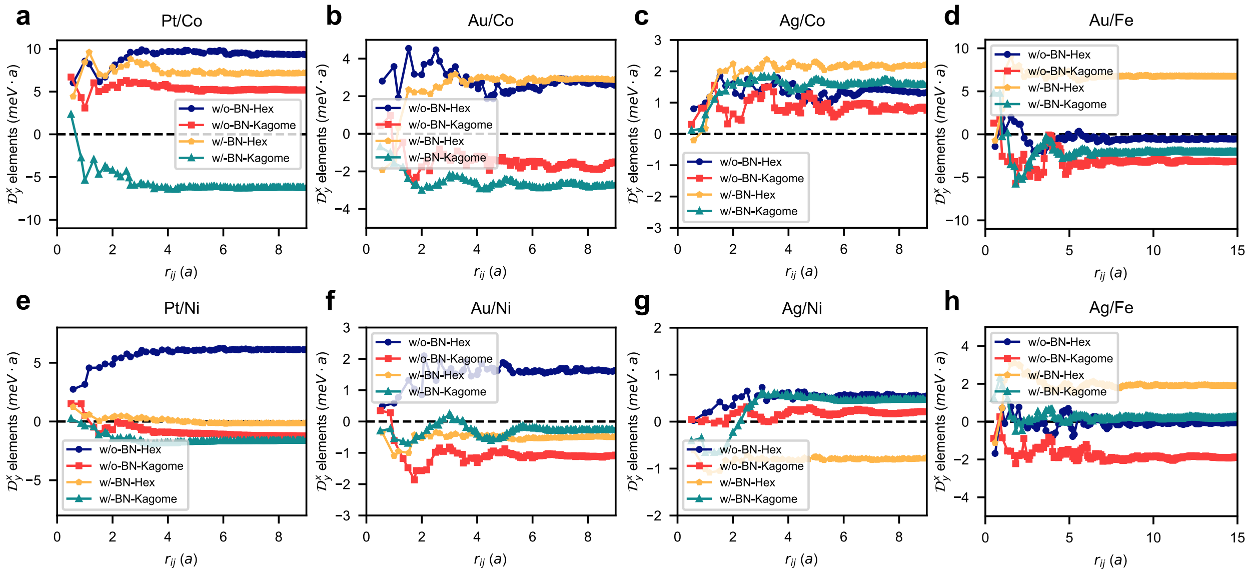

Figure S7: DMI-spiralization-tensor element $\mathcal{D}_y^x$ as a function of the cut-off radius. Panel **a**-**h** are the results for four structural configurations of Pt/Co, Au/Co, Ag/Co, Au/Fe, Pt/Ni, Au/Ni, Ag/Ni, and Ag/Fe systems.

Supplementary Note 6: Tests of exchange and correlation functionals

We are using a well-established methodology for the type of systems that we are addressing. There are two aspects which are difficult to reconcile with the same functional: structural properties and magnetic properties. For the structural properties, the generalized gradient approximation of Perdew, Burke and Ernzerhof (GGA-PBE) is known to give very good results, and so the considered structures were relaxed using the Quantum Espresso package with this functional. For the magnetic properties and magnetic interactions, we used the local spin-density approximation (LSDA) and the all-electron JuKKR computational package. We have also checked the relevance of accounting for van der Waals interactions in the optimized structures. These interactions are more relevant for physisorption (large separation between *h*-BN and the TM layer) than for chemisorption (small separation between those two layers). Regarding magnetic properties, it is known that PBE tends to overestimate the magnetic moments, which affects the magnetic interactions and magnetic anisotropy energy (MAE). We tested the influence of all these aspects for our systems.

In Table S3, we compared the result in the manuscript (PBE+vdW) with those using LSDA, considering the energy differences $\Delta E_{\mathrm{Hex-Kagome}}$ and geometries for three typical examples: Pt/Co/*h*-BN, Au/Ni/*h*-BN and Ag/Ni/*h*-BN. Our results consistently demonstrate that kagome structures are more stable than hexagonal structures across all the functionals we examined, even if in some cases different functionals switch between chemisorption and physisorption (e.g., hexagonal structure of Pt/Co/*h*-BN). Turning to the magnetic properties, we computed the Heisenberg exchange inter-

Pt/Co/*h*-BN		Kagome		Hexagonal	
	$\Delta E_{\mathrm{Hex-Kagome}}$	$z_{\mathrm{BN-Co}}$	$z_{\mathrm{Pt-Co}}$	$z_{\mathrm{BN-Co}}$	$z_{\mathrm{Pt-Co}}$
PBE+vdW	0.51	2.19	2.11	3.13	1.96
LSDA+vdW	1.17	2.02	2.02	2.02	1.86
LSDA	1.26	2.07	2.06	2.18	1.91
Au/Ni/*h*-BN		Kagome		Hexagonal	
	$\Delta E_{\mathrm{Hex-Kagome}}$	$z_{\mathrm{BN-Ni}}$	$z_{\mathrm{Au-Ni}}$	$z_{\mathrm{BN-Ni}}$	$z_{\mathrm{Au-Ni}}$
PBE+vdW	1.35	2.06	2.19	2.21	2.02
LSDA+vdW	1.22	1.94	2.07	1.87	1.85
LSDA	1.35	1.96	2.11	1.93	1.93
Ag/Ni/*h*-BN		Kagome		Hexagonal	
	$\Delta E_{\mathrm{Hex-Kagome}}$	$z_{\mathrm{BN-Ni}}$	$z_{\mathrm{Ag-Ni}}$	$z_{\mathrm{BN-Ni}}$	$z_{\mathrm{Ag-Ni}}$
PBE+vdW	1.03	2.03	2.22	2.36	2.09
LSDA+vdW	1.37	1.90	2.08	1.90	1.90
LSDA	1.30	1.94	2.11	1.94	1.96

Table S3: Energy differences $\Delta E_{\mathrm{Hex-Kagome}}$ (eV) between the hexagonal and the kagome structures of the TM monolayer for the complete structure and geometries (Å) of both kagome and hexagonal structures: vertical BN-TM ($z_{\mathrm{BN-TM}}$) and vertical TM-HM ($z_{\mathrm{TM-HM}}$) distances.

action, Dzyaloshinskii-Moriya interaction (DMI) and MAE of Pt/Co to compare the influence of different functionals on the obtained results in JuKKR code. In Fig S8, we present the micromagnetic parameters (exchange-stiffness-tensor element $\mathcal{A}_{xx}$ and DMI-spiralization-tensor element $\mathcal{D}_y^x$) as a function of the cut-off radius, and we find only minor discrepancies between the two functionals. However, for the MAE we obtained 0.82 meV/Co with LSDA and 0.59 meV/Co with PBE, and this influences the size of the skyrmions we obtained, which change from a diameter of 5 nm (LSDA) to 22 nm (PBE). Here we find the LSDA results more reliable, given our past experience and the cited literature.

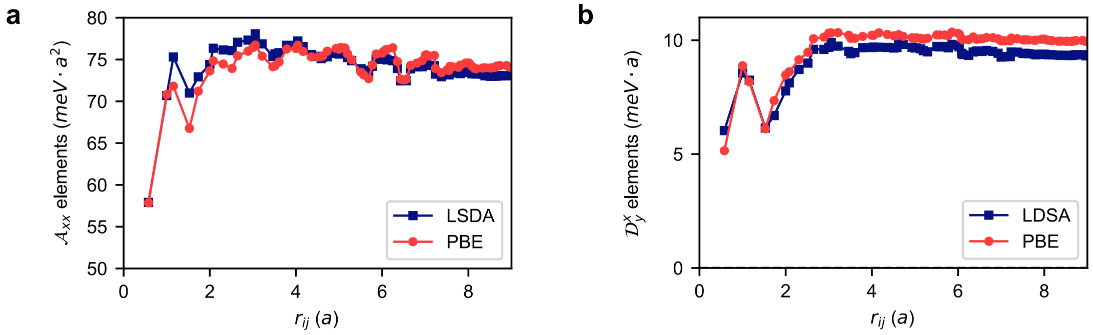

Figure S8: Comparison of magnetic interactions in Pt/Co. **a** Exchange-stiffness-tensor element $\mathcal{A}_{xx}$. **b** DMI-spiralization-tensor element $\mathcal{D}_y^x$.
